# Parker Solar Probe Imaging of the Night Side of Venus

**DOI:** 10.1029/2021GL096302

**Published:** 2022-02-09

**Authors:** Brian E. Wood, Phillip Hess, Jacob Lustig‐Yaeger, Brendan Gallagher, Daniel Korwan, Nathan Rich, Guillermo Stenborg, Arnaud Thernisien, Syed N. Qadri, Freddie Santiago, Javier Peralta, Giada N. Arney, Noam R. Izenberg, Angelos Vourlidas, Mark G. Linton, Russell A. Howard, Nour E. Raouafi

**Affiliations:** ^1^ Naval Research Laboratory Space Science Division Washington DC USA; ^2^ The Johns Hopkins University Applied Physics Laboratory Laurel MD USA; ^3^ Naval Research Laboratory Remote Sensing Division Washington DC USA; ^4^ Facultad de Fisica Universidad de Sevilla Sevilla Spain; ^5^ NASA Goddard Space Flight Center Greenbelt MD USA

## Abstract

We present images of Venus from the Wide‐Field Imager for Parker Solar Probe (WISPR) telescope on board the Parker Solar Probe (PSP) spacecraft, obtained during PSP's third and fourth flybys of Venus on 2020 July 11 and 2021 February 20, respectively. Thermal emission from the surface is observed on the night side, representing the shortest wavelength observations of this emission ever, the first detection of the Venusian surface by an optical telescope observing below 0.8 μm. Consistent with previous observations at 1 μm, the cooler highland areas are fainter than the surrounding lowlands. The irradiances measured by WISPR are consistent with model predictions assuming a surface temperature of *T* = 735 K. In addition to the thermal emission, the WISPR images also show bright nightglow emission at the limb, and we compare the WISPR intensities with previous spectroscopic measurements of the molecular oxygen nightglow lines from Venus Express.

## Key Points

- First results are reported about images of the night side of Venus from the WISPR imager on board Parker Solar Probe
- Thermal emission from the surface is observed at shorter wavelengths than ever before seen
- Nightglow emission from molecular oxygen is observed at the limb

## 1 Introduction

Although Venus is the brightest planet in the sky, its surface was long a mystery due to the opacity of its thick atmosphere. Optical images of the planet show a featureless white disk, dominated by scattered sunlight from the impenetrable atmosphere. Radar imaging from Earth provided the first means to see the surface (e.g., Goldstein et al., 1976; Rogers & Ingalls, 1969), but detailed mapping had to await the arrival of orbiting spacecraft with radar capabilities, particularly Magellan (e.g., Solomon et al., 1992).

Starting with the work of Allen and Crawford (1984), it was found that it was possible to penetrate the thick Venusian atmosphere by observing nightside thermal emissions in the near infrared (NIR), providing a means to study both the surface and lower atmosphere (Kamp et al., 1988; Peralta et al., 2017; Pollack et al., 1993). This has remained an active area of research ever since, involving additional ground‐based measurements (e.g., Arney et al., 2014; de Bergh et al., 1995; Bezard et al., 1990; Crisp et al., 1989; Meadows & Crisp, 1996), observations from Galileo's imaging spectrometer during its 1990 flyby of Venus (e.g., Carlson et al., 1991; Drossart et al., 1993), a NIR spectrum from Cassini during its second flyby in 1999 (Baines et al., 2000), extensive observations from Venus Express during its lengthy 2006–2014 operational lifetime (e.g., Bézard et al., 2009; Helbert et al., 2008; Mueller et al., 2008), and finally measurements from the Akatsuki mission, which is currently still in operation around Venus (e.g., Iwagami et al., 2018; Peralta et al., 2019).

It is at the short wavelength end of the NIR spectral region at 0.8–1.1 μm where the atmospheric opacity is minimized and the surface of the planet is most clearly seen (Peralta et al., 2017). Such observations are therefore most useful for studying the surface emission, potentially providing a means for remotely mapping composition (Baines et al., 2000; Gilmore et al., 2015). However, analyses such as this require maps of the surface across as broad a wavelength range as possible. Extending the thermal emission measurements to shorter wavelengths is difficult, as intensities drop rapidly with decreasing wavelength. To date, the shortest wavelength detection of the emission is from Cassini, which clearly detects emission at 0.85 μm. The Cassini data set consists of only a single 10 s NIR spectrum from the spacecraft's visual‐infrared mapping spectrometer, but that spectrum nevertheless clearly shows the 0.85 *μ*m emission (Baines et al., 2000). This article concerns observations at even shorter wavelengths (<0.80μm) from Parker Solar Probe (PSP), allowing us to extend measurements of the thermal emission into the optical regime.

The PSP observations are from the Wide‐Field Imager for Parker Solar Probe (WISPR) instrument (Vourlidas et al., 2016). This is the only imager on board PSP, which mostly consists of plasma and field instruments devoted to measuring properties of the solar wind close to the Sun. The WISPR instrument consists of a pair of broadband optical telescopes with active‐pixel sensor (APS) detectors, WISPR‐I and WISPR‐O, designed to image the solar wind at different distances from the Sun in the PSP ram direction, both with a bandpass of about 0.5–0.8 *μ*m. Emission from the night side of Venus has previously been observed within this optical bandpass, though not from the surface. Instead, nightglow emission from optical O_2_ lines has been detected, dating back to the Venera 9 and 10 missions (Krasnopolskii et al., 1977; Lawrence et al., 1977). Although observable from the ground (e.g., Slanger et al., 2006), the emission has been most extensively studied using spectra from the Visible and Infrared Thermal Imaging Spectrometer (VIRTIS) on board VEX, particularly at the limb where the emission is at its brightest (García Muñoz et al., 2009, 2013; Migliorini et al., 2013). Besides the O_2_ emission, nightglow from the atomic O I 5577 Å green line has also been frequently observed (Slanger et al., 2006), but this emission is highly variable, and was curiously never detected by VEX. In any case, both the O_2_ and O I nightglow are potentially observable by PSP/WISPR, although O_2_ should always dominate in WISPR's broad bandpass.

## 2 Observations

The PSP mission to explore the solar wind near the Sun began with the launch of the spacecraft on 2018 August 12. A series of seven Venus gravity assist (VGA) flybys are required to push the perihelion of the spacecraft orbit closer to the Sun, with the goal of eventually reaching a perihelion distance of just under 10 R_⊙_ from Sun‐center after the final Venus encounter (VGA7) on 6November 2024. As of this writing, five flybys have occurred so far (VGA1–VGA5). These encounters are providing new information about the planetary environment, particularly its interaction with the solar wind (e.g., Bowen et al., 2021).

The WISPR imager has so far contributed to Venus‐related research through observations of a circumsolar dust ring near the orbit of Venus, based on images acquired far away from the planet itself (Stenborg et al., 2021). As for the flybys, no images were taken by WISPR during VGA1 and VGA2. This changed for VGA3 (11 July 2020) and VGA4 (20 February 2021), providing the first close‐up images of the planet from PSP. Both of WISPR's two telescopes were utilized: WISPR‐I, which observes at elongation angles of 13°–53° from the Sun, and WISPR‐O, which observes at 50°–108° (Vourlidas et al., 2016).

The WISPR telescopes were not designed to study Venus, and there are difficulties using them for this purpose. One problem is that during a flyby the day side of Venus is much too bright for the instrument. The WISPR telescopes have a shutterless design with an effective minimum exposure duration defined by the readout time of about 2 s. This is much too long to observe daylit Venus from up close. Observations from VGA3 and VGA4 demonstrate this explicitly. In images with dayside Venus in the field of view, not only is the planet highly overexposed, but the oversaturation produces scattered light artifacts that contaminate the entire image. Therefore, we expect that the only usable WISPR images taken during any VGA will be ones of the night side, with no part of daylit Venus within the field of view.

The WISPR images of Venus are processed using the standard Level 2 pipeline described in Hess et al. (2021). This processing removes the APS detector column to column bias pattern and corrects and normalizes the images so that the resulting data are consistent, regardless of the exposure time and gain setting used for a given image. The only step in the pipeline not applied to the Venus observations was the calibration coefficient, which is normally used to convert WISPR images from units of digital number per second (DN s^−1^) into mean solar brightness (MSB). The MSB units are useful for comparing WISPR observations to coronal observations from other instruments, but they have little relevance for Venus.

**Figure 1:**
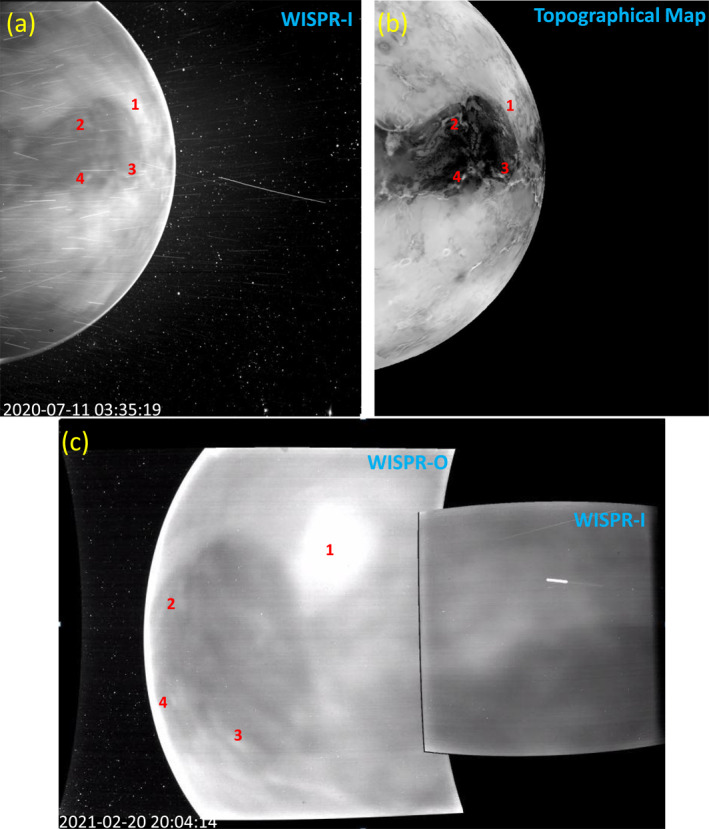
(a) Wide‐Field Imager for Parker Solar Probe‐I (WISPR‐I) image of the nightside of Venus from Venus gravity assist (VGA) 3, showing thermal emission from the surface on the disk and O_2_ nightglow emission at the limb. Black to white represents 0 DN s^−1^ to 40 DN s^−1^ with the scale saturated at 40 DN s^−1^. The image is contaminated by numerous roughly horizontal dust streaks, from material ablating off the Parker Solar Probe heat shield. (b) Topographical map from Magellan, using an inverse black and white scale to match the WISPR image, with bright regions being low elevation and dark regions being high elevation. (c) WISPR‐I and ‐O images of Venus from VGA4. The same part of the Venusian surface is observed as in (a). Red numbers in all panels mark common features for ease of reference. A movie of the VGA4 images is available in the online article.

During VGA3, there were only two usable images taken, both of them by WISPR‐I. The best of these is shown in Figure 1a, which is an 18.4 s exposure from UT 03:35:19. The image contains numerous, mostly horizontal dust streaks, which are often seen in WISPR images, due to material ablating off of PSP's heat shield after impact from interplanetary dust particles. Ignoring the streaks, we clearly see structured emission emanating from the planet, which clearly correlates with topographical surface features. This is shown explicitly by comparing Figures 1a and 1b, the latter being a topographical map of Venus based on version 2 of the Magellan Global Topographic Data Record, with an inverse black and white color scale used for ease of comparison with the WISPR‐I image. It is also instructive to compare Figure 1a with the 1.0 *μ*m Akatsuki image presented by Iwagami et al. (2018; see Figure 17), which happens to be of the same part of the planet. The WISPR‐I and Akatsuki images are both dominated by the dark oval near the equator, which is the Ovda Regio plateau at the western end of Aphrodite Terra, the largest highland region on Venus.

Surface thermal emission is clearly responsible for the disk emission in both the WISPR‐I and 1.0 *μ*m Akatsuki images. As described by Iwagami et al. (2018), the highlands are fainter than the surrounding lowlands due to a temperature difference estimated to be about 30 K. The surface temperature lapse rate for Venus is typically estimated to be about −7 to −8 K/km (Meadows & Crisp, 1996; Seiff et al., 1985). There is one striking difference between the WISPR‐I and 1.0 *μ*m Akatsuki images, and that is that the WISPR‐I image shows a bright rim of emission at the limb of the planet, which is not seen at all at 1.0 *μ*m. We attribute this to the optical O_2_ nightglow. A similar rim of limb‐brightened nightglow emission is seen in nightside images from the Venus Monitoring Camera (VMC) instrument on VEX using the VIS filter (roughly 480–600 nm), with no detectable emission from the disk (García Muñoz et al., 2013).

The experience of the VGA3 observations helped inform the planning for VGA4, which allowed for better viewing of the Venusian nightside. As a result, VGA4 was much more productive than VGA3, providing a much larger collection of usable WISPR exposures, involving both WISPR‐I and WISPR‐O. The VGA4 images are also relatively free of the dust streaks that affect VGA3. An example of one of the VGA4 images is illustrated in Figure 1c, showing the overlapping fields of view of WISPR‐I on the right and WISPR‐O on the left. The WISPR‐I and WISPR‐O images have exposure times of 2.82 and 3.84 s, respectively. In contrast to VGA3, during VGA4, PSP passed in front of Venus in its orbit instead of behind, so the WISPR images track the western limb of the planet instead of the eastern limb. A movie covering the full sequence of images is available in the online version of this article. By chance, the part of the Venusian surface on the night side is roughly the same for VGA3 and VGA4, so Ovda Regio is once again particularly prominent, filling much of the WISPR‐O field of view in Figure 1c. Common features within the VGA3 and VGA4 images are illustrated in the figure. In the VGA4 movie, these features can be seen moving from the WISPR‐O field of view into that of WISPR‐I as PSP passes behind the planet, with the sunrise for the spacecraft approaching the western limb in the WISPR‐I field of view at the end of the movie.

**Figure 2:**
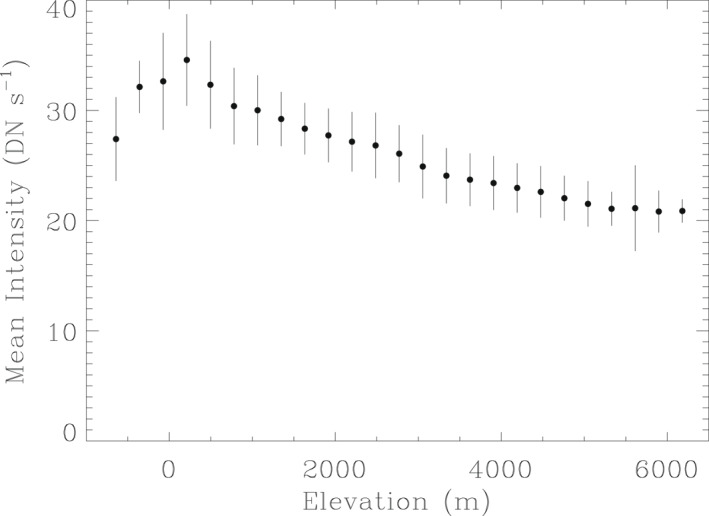
Mean count rate per pixel digital number per second in the Wide‐Field Imager for Parker Solar Probe‐O image from Figure 1(b), plotted versus elevation. The error bars indicate 1*σ* standard deviations. The decrease in intensity with increasing altitude is due to a decrease in surface temperature.

In order to explicitly illustrate the observed intensity dependence on elevation, we first identify the elevation of each pixel in the WISPR‐O image from Figure 1c using the topographical map of Venus (see Figure 1b). The individual pixel count rates in DN s^−1^ are placed in altitude bins, and we then plot in Figure 2 the mean and standard deviation of the intensities as a function of altitude, relative to a reference radius of 6,051 km. The decrease in brightness with altitude is clearly seen. The anomalous behavior seen for the lowest altitudes is due to these points being almost entirely from one region, in the far upper right corner of the WISPR‐O image in Figure 1c, which is apparently somewhat darker than the general trend would predict.

It is worth noting that the standard deviations in Figure 1 are indicative of real variance, and are not dominated by noise in the data or systematic uncertainties in the images. We have confirmed this by comparing successive images from the VGA4 data set, to verify that points on the surface maintain consistent intensities despite shifting positions on the detector. The variances seen in Figure 2 will be due to many factors such as atmospheric blurring, contamination by scattered solar light, contamination by disk nightglow emission, spatial variations in atmospheric opacity, and potentially mineralogical variations. Some of these issues will be discussed further in the next section.

## 3 Measurements

We have identified two sources of emission in the WISPR images of Venus, surface thermal emission on the disk and O_2_ nightglow emission at the limb. We now seek to assess whether our observed intensities are consistent with expectations.

We focus first on the surface thermal emission. Considering that intensities should be over two magnitudes higher at 1 *μ*m than within WISPR's nominal bandpass below 0.8 *μ*m, one concern is whether a red leak might exist for the WISPR optics that could account for the detected emission. Additional laboratory measurements were made using analogs of those optics in order to look for any indication of throughput above 0.8 *μ*m, but no evidence for such a leak was found.

**Figure 3:**
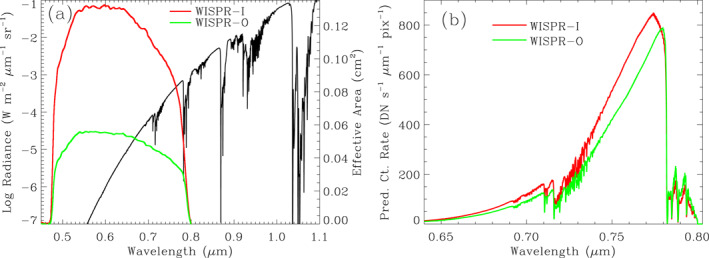
(a) A model spectrum of the surface thermal emission from Venus, assuming a temperature of 735 K (left axis). Also shown are the effective area curves of Wide‐Field Imager for Parker Solar Probe (WISPR) WISPR‐I and WISPR‐O (right axis). (b) Count rates per pixel for WISPR‐I and WISPR‐O are predicted by the model spectrum from (a).

Further evidence that we are seeing thermal surface‐emission within WISPR's optical bandpass comes from demonstrating that the WISPR count rates are consistent with model predictions. Figure 3a shows a normal‐incidence model spectrum of thermal emission from the Venusian surface, assuming a temperature of 735 K, which should apply at an altitude of 0 km (Seiff et al., 1985). The spectral model was produced using the line‐by‐line, multi‐stream, multi‐scattering spectral mapping atmospheric radiative transfer code (Meadows & Crisp, 1996). The Venus International Reference Atmosphere was used for the vertical thermal profile and atmospheric composition (Moroz & Zasova, 1997), with updates to the lower atmosphere from Arney et al. (2014). Sulfuric acid clouds were simulated using the model from Crisp (1986). Rotational‐vibrational molecular absorption coefficients were calculated using the line‐by‐line absorption coefficient code (Crisp, 1997; Meadows & Crisp, 1996), with inputs from HITRAN2016 (Gordon et al., 2017). We assumed surface albedo and emissivity curves consistent with a basaltic surface. Thermal radiance spectra were produced using 8‐stream calculations at 1 cm^−1^ wavenumber resolution. The model reproduces observed intensities at 1.0 μm (e.g., Meadows & Crisp, 1996), providing confidence in its ability to approximate intensities at lower wavelengths as well.

Multiple molecular absorption bands are observed in the model spectrum. The ones within the WISPR bandpass are near 0.72 and 0.79 *μ*m. The 0.72 *μ*m band is a blend of absorption from CO_2_ and H_2_O, while the 0.79 *μ*m band is entirely CO_2_. In addition to this discrete absorption, the model spectrum exhibits a broadband attenuation of about a factor of 4 due to absorption from the sulfuric acid clouds. The opacity of the thick atmosphere leads to significant blurring. This effect is presumably why the WISPR images do not show a finer scale structure.

Multiplying the model spectrum in Figure 3a by the WISPR effective area curves shown in the figure yields predictions for WISPR count rates per pixel. Taking into account pixel sizes of 1.26′ (or 1.34 × 10^−7^ sr) and 1.71′ (or 1.82 × 10^−7^ sr) for WISPR‐I and ‐O, respectively, and a gain value of 2.716 e^−^/DN, we derive the count rate spectra for WISPR‐I and ‐O shown in Figure 3b. Integrating over wavelength, the count rates predicted for WISPR‐I and ‐O are 40.0 DN s^−1^ and 33.5 DN s^−1^, respectively. The 33.5 DN s^−1^ WISPR‐O prediction is in very good agreement with the zero‐altitude value displayed in Figure 2, which is encouraging since the assumed *T* = 735 K temperature of the model spectrum is the presumed zero‐altitude temperature (Seiff et al., 1985).

Figure 3b indicates the wavelength distribution of the photons that WISPR is seeing, based on the model spectrum. We are seeing the Venusian surface right at the ill‐defined boundary between the optical and NIR spectral regimes. This optical/NIR boundary is often quoted to be at 0.75 *μ*m. Although most of the flux observed by WISPR is predicted to be coming from just above 0.75 *μ*m, there is still a significant contribution (∼39%) from lower wavelengths, consistent with our characterization of the WISPR observations as the first‐ever optical images of the Venusian surface. The spectral location of the thermal emission far from the center of WISPR's broad bandpass is the primary reason why we forward the model all the way to DN before comparing with the data rather than trying to convert the images from DN to radiance. Such a conversion would require an a priori assumption of some spectral shape, which is very different for the thermal and O_2_ emission, which in turn are very different from the scattered solar light that WISPR more commonly studies.

We now turn our attention to limb emission. This emission is highest near the equator and decreases toward higher northern and southern latitudes, as best seen in the VGA3 image in Figure 1a. This latitudinal behavior is consistent with that observed by VEX/VMC (García Muñoz et al., 2013). Using the images in Figure 1 as a representative, we find a typical limb brightness near the equator to be about 80 DN s^−1^, for both the WISPR‐I and WISPR‐O images in panels (a) and (c), respectively. We compute auroral intensities from these values using the previously mentioned gain values and response curves and assuming the VEX/VIRTIS O_2_ nightglow spectrum from García Muñoz et al. (2009) as a guide for the flux distribution amongst the various O_2_ lines. We estimate auroral intensities in kilo‐Rayleigh units of 239 and 285 kR for WISPR‐I and WISPR‐O, respectively. These intensities are within the range of values observed by VEX, although near the upper end of that range, with VEX more typically seeing intensities of 150 kR at low latitudes (García Muñoz et al., 2013). It is possible that the nightglow was simply brighter during PSP's recent visits to Venus, but the possibility of scattered light contributing to the signal should also be considered. The thick Venusian atmosphere is effective at refracting sunlight from the dayside to the nightside. Contributions from this scattered light are evident in VEX images (García Muñoz et al., 2013; Longobardo et al., 2012; Mueller et al., 2008). Scattered light could be even stronger at the shorter wavelengths observed by WISPR. The VGA4 data set can be used to study the dependence of the emission on solar phase angle, which should allow an assessment of the contribution of scattered light to the signal, but we reserve this more detailed analysis for a future study.

One issue that should be addressed concerns contamination of the thermal emission on the disk with the O_2_ nightglow emission. We only see the O_2_ emission clearly at the limb due to dramatic limb brightening, but there should be emission on the disk at some level, although VEX/VMC failed to detect it. Experience with the much brighter O_2_ line at 1.27 *μ*m suggests a factor of 50 difference in intensity between the limb and the disk (Gérard et al., 2008). This ratio would predict a count rate of about 1.6 DN s^−1^ on the disk, representing a contribution of order 5% to the disk emission.

## 4 Conclusions

The PSP/WISPR observations have provided the first images of the Venusian surface in the optical, but there have actually been many reports of faint emission from the Venusian night side from credible amateur and professional astronomers, dating back to the 1600s (Baum, 2000; Benton, 2018; McKim, 2019). This “ashen light” phenomenon, as it has come to be called, has never been successfully imaged, however, leading to the suspicion that the phenomenon may be an optical illusion (Sheehan et al., 2014). It would be a worthwhile project for both amateur and professional astronomers to assess whether the optical surface thermal emission seen by PSP/WISPR might be sufficiently bright to be observable from the ground. One fundamental difficulty with ground‐based observations is the blinding presence of a daylit crescent Venus, which is always there. The excellent dynamic range of the human eye might give the eye an advantage over electronic detectors in discerning something very faint near something so bright, but only reproducible images can provide a truly convincing detection.

Observers using only the eye to view the Venusian night side, rather than an electronic detector, should be aware that although we have estimated the O_2_ nightglow to be only about a 5% contributor on the disk in the WISPR images, this will not be true for the human eye. For the eye, it is the O_2_ nightglow that seems likely to be the dominant source of disk emission. Unlike WISPR, the eye is roughly three orders of magnitude less sensitive at the ∼0.75μm wavelength of the detected thermal emission than the 0.45–0.55 *μ*m wavelengths of the strongest O_2_ lines.

The future of Venusian surface and nightside studies looks bright due to the recent initiation of NASA and ESA support for three brand new missions to Venus. The two new NASA missions are VERITAS and DAVINCI+, and the new ESA mission is EnVision. All of these spacecraft are currently planned to carry NIR imaging capabilities, allowing the study of the nightside surface. With WISPR having demonstrated the possibility of extending surface observations into the optical, it would be prudent to explore the diagnostic power of these shorter wavelengths. As for future PSP/WISPR observations, unfortunately the encounter geometry for the very recent VGA5 flyby in 2021 October was not favorable for nightside imaging, and the VGA6 encounter in 2023 August will be no better. Thus, we will probably have to await the final flyby (VGA7) on 6 November 2024 for the new WISPR imagery of Venus.

## Supporting information

Movie S1

## Data Availability

The Venus Magellan Global Topography 4641m v2 data set used for elevation information was obtained from the Planetary Data System. The official gateway for PSP data is https://sppgway.jhuapl.edu, with the WISPR images studied here specifically located at https://wispr.nrl.navy.mil/data/rel/fits/CAL1/20 200 711/ and https://wispr.nrl.navy.mil/data/rel/fits/L1/highcadence/20 210 220/.
